# (C_5_H_6.16_N_2_Cl_0.84_)(IO_2_Cl_2_): a birefringent crystal featuring unprecedented (IO_2_Cl_2_)^−^ anions and π-conjugated organic cations†

**DOI:** 10.1039/d3sc05770d

**Published:** 2023-11-27

**Authors:** Qian-Qian Chen, Chun-Li Hu, Ming-Zhi Zhang, Jiang-Gao Mao

**Affiliations:** a State Key Laboratory of Structural Chemistry, Fujian Institute of Research on the Structure of Matter, Chinese Academy of Sciences Fuzhou 350002 P. R. China mjg@fjirsm.ac.cn; b University of Chinese Academy of Sciences Beijing 100039 P. R. China

## Abstract

Birefringent crystals can manipulate the polarization state of lasers and have vital application in polarizers, optical isolators, phase compensators, *etc.* The design and synthesis of crystals with large birefringence remains a challenging task. To design crystals with large birefringence, we combine an unprecedented chloroiodate(v) group (IO_2_Cl_2_)^−^ featuring large polarizability anisotropy and a strong stereochemically active lone pair (SCALP) with the π-conjugated 2-amino-5-chloropyridine group. The superior synergy effect of (IO_2_Cl_2_)^−^ and 2-amino-5-chloropyridine groups produces a new birefringent crystal, namely (C_5_H_6.16_N_2_Cl_0.84_)(IO_2_Cl_2_). It exhibits remarkably large birefringence of 0.67 at 546 nm, far exceeding those of most visible birefringent materials reported. This work discovers the first chloroiodate(v) group and provides a new synthetic route for birefringent materials.

## Abstract 2

(C_5_H_6.16_N_2_Cl_0.84_)(IO_2_Cl_2_) composed of unprecedented (IO_2_Cl_2_)^−^ anions and π-conjugated (C_5_H_6.16_N_2_Cl_0.84_)^+^ cations exhibits enormous birefringence of 0.67 at 546 nm.

## Graphical abstract

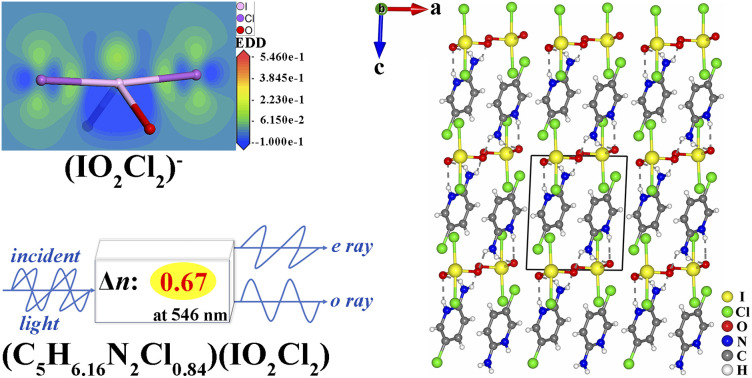

## Introduction

Birefringent crystals could modulate the polarization-dependent light propagation, and they are widely applied in optical isolators, polarizers, and phase compensators.^1–6^ The crystals with giant optical anisotropy could effectively manipulate light and miniaturize the fabricated devices.^6^ In the visible region, the materials show relatively small birefringence (below 0.3) such as commercialized YVO_4_ (Δ*n*_exp._: 0.204 at 532 nm) and CaCO_3_ (Δ*n*_exp._: 0.172 at 589 nm).^7,8^ Only a few crystals exhibit birefringence greater than 0.5, such as Cd(H_2_C_6_N_7_O_3_)_2_·8H_2_O (Δ*n*_exp._: 0.60 at 550 nm) and SnPO_4_I (Δ*n*_exp._: 0.664 at 546 nm).^9–11^ However, Cd(H_2_C_6_N_7_O_3_)_2_·8H_2_O suffers from low thermal stability (85 °C), and SnPO_4_I (band gap: 2.45 eV) shows a limited visible transparency window. The birefringence of Cd(H_2_C_6_N_7_O_3_)_2_·8H_2_O and SnPO_4_I arises from the π-conjugated (H_2_C_6_N_7_O_3_)^−^ groups and SCALP Sn(ii)-based groups, respectively. Numerous studies illustrate that π-conjugated groups and SCALP groups are efficient birefringent-active groups, and incorporating these groups regularly can result in large birefringence.^4,10–14^

Besides the Sn(ii)-based materials, metal iodates containing strong SCALPs are also a class of promising visible birefringent crystals.^15,16^ The birefringence of most iodates is in the range of 0.05–0.25 as exemplified by CsIO_3_ (Δ*n*_cal._: 0.19 at 1064 nm).^17–20^ Partial substitution of oxygen atoms with fluorine atoms afforded fluoroiodate groups such as (IO_3_F)^2−^ and (IO_2_F_2_)^−^, which show larger polarizability anisotropy than IO_3_^−^ groups.^21^ About 83% of the reported fluoroiodates are assembled from (IO_2_F_2_)^−^ groups but show relatively small birefringence (Δ*n*: 0.05–0.20) as exemplified by CsIO_2_F_2_ (Δ*n*_cal._: 0.046 at 1064 nm).^19–22^ The large electronegativity of F causes the F-2p states to locate at a low energy, usually playing a negative role in birefringence.^22–24^ To get out of the dilemma, we select the Cl atom with lower electronegativity to design the chloroiodate(v) group (IO_2_Cl_2_)^−^. According to our calculations, the large difference in I–Cl and I–O bond lengths of (IO_2_Cl_2_)^−^ groups generates polarizability anisotropy which is about five times larger than that of (IO_3_)^−^ and (IO_2_F_2_)^−^ groups. The strength of SCALPs on (IO_2_Cl_2_)^−^ groups would be enhanced due to the strong interaction of I-5s5p and Cl-3p states near the Fermi level, favouring large birefringence.^11–14^ However, no chloroiodate(v) has been reported so far, probably due to the difficulty of replacing strong I–O bonds with weak I–Cl bonds. The (IO_2_Cl_2_)^−^ was only reported theoretically as a transient intermediate, reflecting its possible existence.^25^

π-conjugated groups possessing delocalized p_π_ electrons and prominent polarizability anisotropy can also serve as perfect birefringent-active groups, such as NO_3_^−^, (B_3_O_6_)^3−^, (H_2_C_6_N_7_O_3_)^−^, (H_*x*_C_3_N_3_O_3_)^(3−*x*)−^, (H_*x*_C_6_N_9_)^(3−*x*)−^ (*x* = 0, 1, 2), [C(NH_2_)_3_]^+^ and (C_5_H_6_NO)^+^.^10,15,26–33^ The introduction of π-conjugated groups into metal iodates can enhance birefringence significantly, as exemplified by Sc(IO_3_)_2_(NO_3_) (Δ*n*_exp._: 0.348 at 546 nm).^15,28,33^ Herein we select the π-conjugated 2-amino-5-chloropyridine group featuring a push–pull electronic structure and intramolecular charge transfer, which can promote p_π_ electron delocalization and polarizability anisotropy.^34^ The combination of SCALP chloroiodate(v) groups and π-conjugated 2-amino-5-chloropyridine groups led to a new birefringent crystal, namely, (C_5_H_6.16_N_2_Cl_0.84_)(IO_2_Cl_2_). It shows enormous birefringence (Δ*n*_exp._: 0.67 at 546 nm), exceeding those of most visible birefringent materials reported.

## Results and discussion

Crystals of (C_5_H_6.16_N_2_Cl_0.84_)(IO_2_Cl_2_) were successfully grown by the evaporation method in aqueous hydrochloric acid media (see ESI†). The (IO_2_Cl_2_)^−^ group is formed at a high HCl/HIO_3_ molar ratio of 3 : 1 due to the much stronger I–O bonds compared with I–Cl bonds. Under such conditions, one H atom on the meta position of 2-aminopyridine is partially replaced by the Cl(3) atom with a Cl/H ratio of 0.84 : 0.16, leading to the formation of the (C_5_H_6.16_N_2_Cl_0.84_)^+^ cation, which is similar to the conversion of 2-aminopyridine to 2-amino-5-chloropyridine (see ESI†).^35^

The crystals of (C_5_H_6.16_N_2_Cl_0.84_)(IO_2_Cl_2_) are shown in Fig. S1.† Its purity was confirmed by powder X-ray diffraction (Fig. S2, ESI†). The results of elemental analysis are close to the calculated values (see ESI†). The existence of I and Cl was confirmed by energy-dispersive X-ray spectroscopy (Fig. S3 and S4, ESI†). (C_5_H_6.16_N_2_Cl_0.84_)(IO_2_Cl_2_) crystallizes in the triclinic space group *P*-1 (no. 2) (Tables S1–S4, ESI†). Its asymmetric unit consists of one (C_5_H_6.16_N_2_Cl_0.84_)^+^ cation and one (IO_2_Cl_2_)^−^ anion, and all of the atoms are located at the general sites. In the (C_5_H_6.16_N_2_Cl_0.84_)^+^ cation, the bond lengths of C–C, C–N, and C–Cl are in the ranges of 1.344(5)–1.410(5) Å, 1.325(4)–1.360(4) Å, and 1.715(4) Å, respectively. The I(1) atom is four-coordinated by two O atoms and two Cl atoms to form a seesaw-shaped (IO_2_Cl_2_)^−^. The two I–Cl bond lengths (2.4929(8) and 2.4963(8) Å) are much longer than the two I–O bond lengths (1.784(2) and 1.791(2) Å), and the Cl–I–Cl and O–I–O bond angles are 172.75(3)° and 99.6(1)°, respectively. These I–O and I–Cl bond lengths are close to those of IO_3_^−^ groups (1.73(3)–1.89(2) Å) and (ICl_4_)^−^ groups (2.497(1)–2.515(1) Å) reported previously.^17–20,36^ The calculated bond valence value of I(1) is 5.048, confirming its oxidation state of +5.^37^

**Fig. 1:**
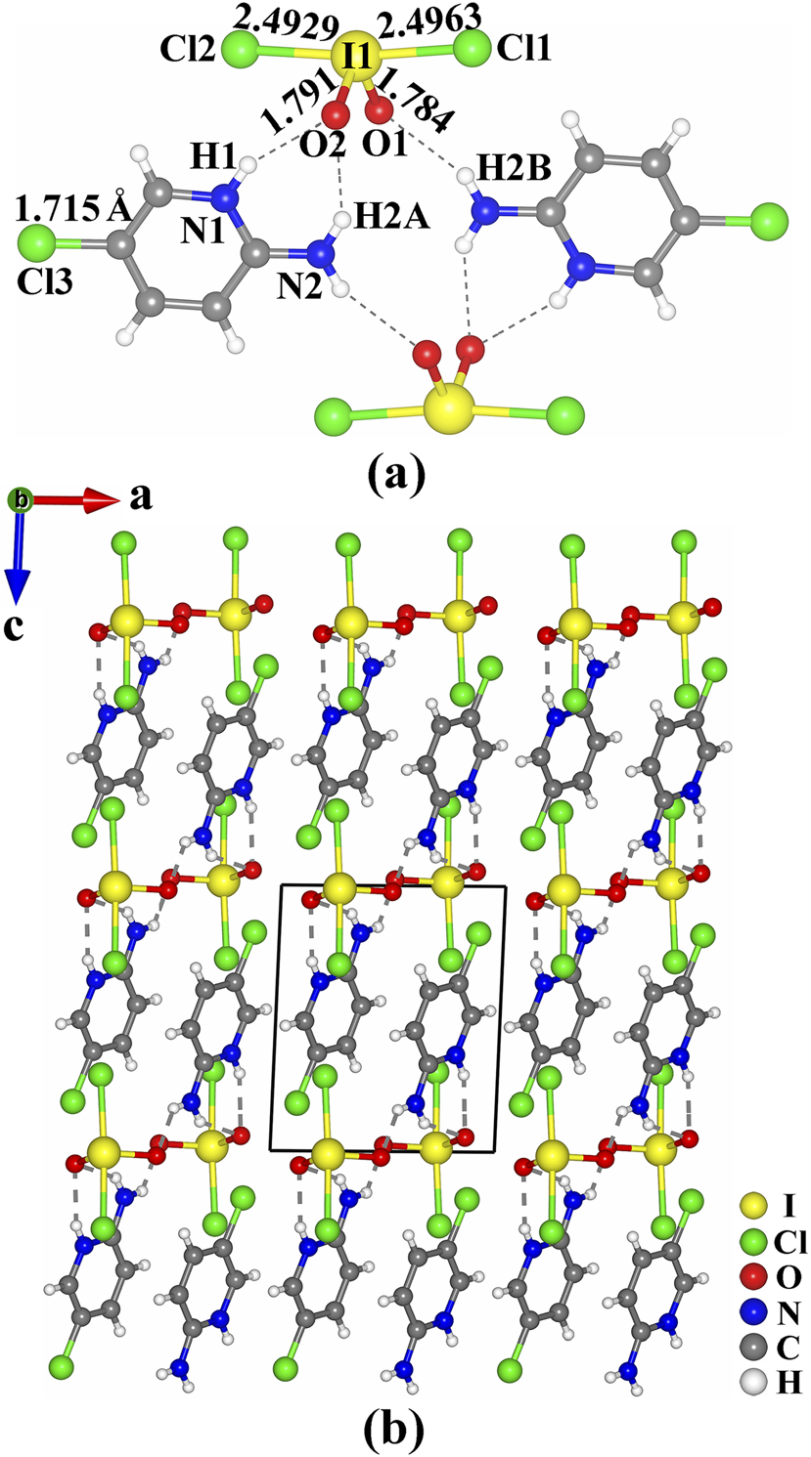
Views of the [(C_5_H_6.16_N_2_Cl_0.84_)(IO_2_Cl_2_)]_2_ dimeric unit (a), and the structure of (C_5_H_6.16_N_2_Cl_0.84_)(IO_2_Cl_2_) (b).

In (C_5_H_6.16_N_2_Cl_0.84_)(IO_2_Cl_2_), two (C_5_H_6.16_N_2_Cl_0.84_)^+^ cations and two (IO_2_Cl_2_)^−^ anions are interconnected through hydrogen bonds (N(1)–H(1)⋯O(2) 2.875(3) Å, N(2)–H(2A)⋯O(2) 2.999(4) Å and N(2)–H(2B)⋯O(1) 2.994(4) Å) into a [(C_5_H_6.16_N_2_Cl_0.84_)(IO_2_Cl_2_)]_2_ dimeric unit (Fig. 1a). These dimeric units are interlinked into a three-dimensional (3D) supramolecular structure through π–π stacking interactions with an inter-ring distance of 3.75 Å between neighboring parallel (C_5_H_6.16_N_2_Cl_0.84_)^+^ cations (Fig. 1b).

Thermogravimetric analysis demonstrated that (C_5_H_6.16_N_2_Cl_0.84_)(IO_2_Cl_2_) is thermally stable up to 110 °C, after which it is totally decomposed in one step from 110 °C to 1000 °C (Fig. S5, ESI.† The infrared (IR) spectrum is shown in Fig. S6† and the detailed assignments of the IR bands are presented in Table S5.† (ref. 38) The peaks located at 890–650 cm^−1^ signify the I–Cl and I–O stretching vibrations, and the absorption bands observed at 650–400 cm^−1^ correspond to the I–Cl and I–O bending vibrations. The UV-vis-NIR diffuse reflectance spectrum revealed its UV absorption cutoff edge of 325 nm, and its band gap of 3.38 eV (Fig. S7, ESI†). The band gap is larger than those of some outstanding birefringent materials such as Sn_2_PO_4_I (2.45 eV) and (C_3_N_6_H_8_)PbBr_4_ (3.13 eV), but is smaller than those of some pyridine iodates, fluoroiodates and iodate chlorides such as [*o*-C_5_H_4_NHOH]_2_[I_7_O_18_(OH)]·3H_2_O (3.90 eV), CsIO_2_F_2_ (4.5 eV) and Ba(IO_3_)Cl (4.32 eV).^11,20,28,39,40^ Under the excitation at 365 nm, (C_5_H_6.16_N_2_Cl_0.84_)(IO_2_Cl_2_) displays a blue emission band maximizing at 410 nm (Fig. S8, ESI†), but its photoluminescence quantum yield is less than 1%. A significant blue shift relative to the neutral 2-amino-5-chloropyridine is observed probably due to the protonation of the organic molecule and the effect of (IO_2_Cl_2_)^−^ on its HOMO and LUMO.^41^

**Fig. 2:**
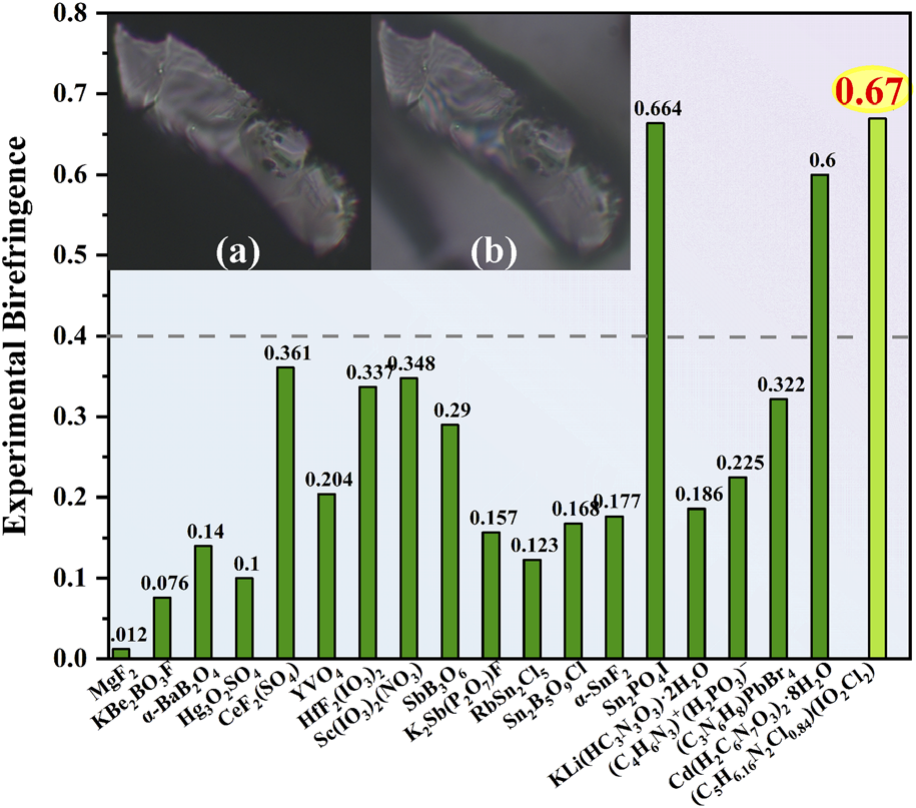
Comparison of birefringence of some excellent birefringent materials at 514–550 nm. The embedded graphs: the original crystal of (C_5_H_6.16_N_2_Cl_0.84_)(IO_2_Cl_2_) under the cross-polarized light (a), and the crystal achieving complete extinction (b).

Based on the polarizing microscope method, the birefringence of (C_5_H_6.16_N_2_Cl_0.84_)(IO_2_Cl_2_) was measured at *λ* = 546 nm using the (010) crystal plane (Fig. 2 and S9, S10†). The optical path difference is 15172.51 nm, and the thickness of the measured crystal is 22.7 μm. The experimental birefringence of (C_5_H_6.16_N_2_Cl_0.84_)(IO_2_Cl_2_) is 0.67 at 546 nm. Its birefringence obviously exceeds those of most visible birefringent materials reported, such as CsIO_3_ (Δ*n*_cal._: 0.19 at 1064 nm), RbIO_2_F_2_ (Δ*n*_cal._: 0.058 at 1064 nm), SrI_2_O_5_F_2_ (Δ*n*_cal._: 0.203 at 532 nm), Ba(IO_3_)Cl (Δ*n*_cal._: 0.118 at 1064 nm), Rb_2_HC_3_N_3_O_3_ (Δ*n*_cal._: 0.4 at 532 nm), Cd(H_2_C_6_N_7_O_3_)_2_·8H_2_O (Δ*n*_exp._: 0.60 at 550 nm), and Sc(IO_3_)_2_(NO_3_) (Δ*n*_exp._: 0.348 at 546 nm).^8,10–22,30–32,39,40,42–51^

**Fig. 3:**
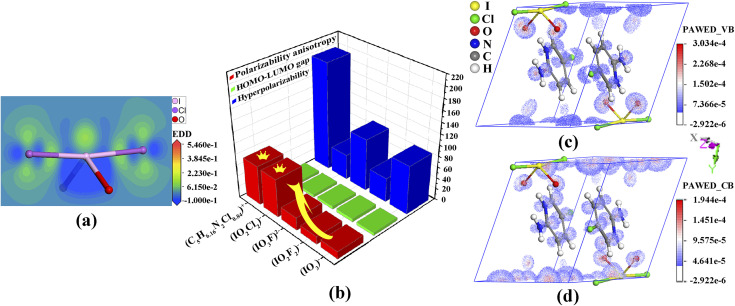
Electron density difference map of the (IO_2_Cl_2_)^−^ group (a), some properties of the related groups (b), and the polarizability anisotropy-weighted electron density in the VB (c) and CB (d) for (C_5_H_6.16_N_2_Cl_0.84_)(IO_2_Cl_2_).

Band-structure calculations *via* the density functional theory indicated that (C_5_H_6.16_N_2_Cl_0.84_)(IO_2_Cl_2_) has a direct band gap of 2.94 eV, which is smaller than the experimental band gap of 3.38 eV (Fig. S11, ESI†). The scissor operator of 0.44 eV was applied for calculating birefringence. In the partial density of states (PDOS) diagram (Fig. S12, ESI†), the topmost valence band (VB) and the bottommost conduction band (CB) are mainly comprised of C-2p and N-2p states. Hence, the band gap of (C_5_H_6.16_N_2_Cl_0.84_)(IO_2_Cl_2_) is dominated by (C_5_H_6.16_N_2_Cl_0.84_)^+^. In the PDOS, the strong interaction of I-5s5p and Cl-3p states near the Fermi level is beneficial for the strength of SCALPs on I^5+^, which is positively related to the birefringence.^11–14^ The SCALP of the (IO_2_Cl_2_)^−^ group could be visually comprehended from an electron density difference (EDD) map (Fig. 3a). The seesaw-shaped (IO_2_Cl_2_)^−^ group could be also seen as a trigonal bipyramid with one of the trigonal vertexes occupied by the SCALP.

Under *λ* = 546 nm, the calculated refractive index features *n*_*z*_ ≫ *n*_*y*_ > *n*_*x*_ along the principal dielectric axes (Fig. S13, ESI†). The angles between the crystallographic axes and the principal dielectric axes are shown in Table S6 (ESI†). The calculated birefringence of (C_5_H_6.16_N_2_Cl_0.84_)(IO_2_Cl_2_) is 0.67 at 546 nm, which matches with the experimental result.

The mechanism of birefringence is analysed below. First, the (C_5_H_6.16_N_2_Cl_0.84_)^+^ and (IO_2_Cl_2_)^−^ groups display large polarizability anisotropies of 71.1 and 69.3, respectively, which are approximately five times greater than those of IO_3_^−^ and IO_2_F_2_^−^ groups (Fig. 3b). Second, the (C_5_H_6.16_N_2_Cl_0.84_)^+^ groups are aligned perfectly in parallel and deviate from the (100) crystal plane with a dihedral angle of 43.8°. Thus, Δ*n* = *n*_in-plane_ − *n*_out-plane_ = *n*(100) − *n*_*x*_.^10^ Third, from the EDD map (Fig. S14, ESI†), the SCALPs manifest regular arrangements in the direction deviating from the *y*-axis by 18.9°, and the polarizable I–Cl bonds deviate from the *z*-axis by 5.6°, which leads to *n*_*z*_ ≫ *n*_*y*_.^13,52^ So, Δ*n* = *n*(100) − *n*_*x*_ = *n*_*z*_ − *n*_*x*_ = *n*(010). The (IO_2_Cl_2_)^−^ and (C_5_H_6.16_N_2_Cl_0.84_)^+^ groups are both located in suitable locations and fastened by hydrogen bonds, which prominently enhances the (010) in-plane anisotropy and thus leads to the large birefringence.^52^

To intuitively unveil the source of birefringence of (C_5_H_6.16_N_2_Cl_0.84_)(IO_2_Cl_2_), polarizability anisotropy-weighted electron density (PAWED) is displayed in Fig. 3c and d. In the VB, the nonbonding Cl-3p and O-2p of (IO_2_Cl_2_)^−^, and the p states in (C_5_H_6.16_N_2_Cl_0.84_)^+^ play a pivotal role in enhancing birefringence. In the CB, the birefringence chiefly originates from the unoccupied I-5p along with Cl-3p and O-2p states of (IO_2_Cl_2_)^−^, and the C-2p and N-2p states in (C_5_H_6.16_N_2_Cl_0.84_)^+^. Thus, the exceptional birefringence is synergistically determined by (IO_2_Cl_2_)^−^ and (C_5_H_6.16_N_2_Cl_0.84_)^+^ groups with the precise contribution values of 62.9% and 37.1%, respectively. This also demonstrates that the (IO_2_Cl_2_)^−^ group is a highly effective birefringent-active group.

## Conclusions

In summary, we have isolated a new birefringent crystal (C_5_H_6.16_N_2_Cl_0.84_)(IO_2_Cl_2_) composed of unprecedented (IO_2_Cl_2_)^−^ anions and π-conjugated (C_5_H_6.16_N_2_Cl_0.84_)^+^ cations. It displays very large birefringence of 0.67 at 546 nm, and thus it is a new candidate for visible birefringent materials. This work initiates research on the chloroiodate(v) system and affords a new route for the design of excellent birefringent materials.

## Author contributions

Qian–Qian Chen: conceptualization, data curation, methodology, visualization, writing – original draft; Chun-Li Hu: formal analysis; Ming-Zhi Zhang: data curation; Jiang-Gao Mao: conceptualization, writing – review & editing, supervision.

## Conflicts of interest

There are no conflicts to declare.

## Supplementary Material

SC-014-D3SC05770D-s001

SC-014-D3SC05770D-s002
