# Supplementary material for: (C5H6.16N2Cl0.84)(IO2Cl2): a birefringent crystal featuring unprecedented (IO2Cl2)− anions and π-conjugated organic cations
Source: Chem Sci. 2023 Nov 27;14(48):14302–7. doi: 10.1039/d3sc05770d (PMC10718177; doi:10.1039/d3sc05770d)
Supplement: SC-014-D3SC05770D-s001 [file SC-014-D3SC05770D-s001.pdf]

Electronic Supplementary Material (ESI) for Chemical Science.
This journal is © The Royal Society of Chemistry 2023

Supporting Information

$(C_5H_{6.16}N_2Cl_{0.84})(IO_2Cl_2)$: a birefringent crystal featuring unprecedented $(IO_2Cl_2)^-$ anions and π-conjugated organic cations

Qian-Qian Chen,[a,b] Chun-Li Hu,[a] Ming-Zhi Zhang[a,b] and Jiang-Gao Mao[a,b]*

[a] State Key Laboratory of Structural Chemistry, Fujian Institute of Research on the Structure of Matter, Chinese Academy of Sciences, Fuzhou 350002, P. R. China.

[b] University of Chinese Academy of Sciences, Beijing 100039, P. R. China.

Email: mjg@fjirsm.ac.cn

Table of Contents

Section	Title	Page
Experimental procedures	Instruments and property characterizations; Synthesis; Single crystal structure determination; Computational methods.	S3-4
Table S1	Crystallographic data for $(C_5H_{6.16}N_2Cl_{0.84})(IO_2Cl_2)$.	S5
Table S2	Selected bond lengths (Å) for $(C_5H_{6.16}N_2Cl_{0.84})(IO_2Cl_2)$.	S6
Table S3	Atomic coordinates (×10^4), equivalent isotropic displacement parameters (Å^2 ×10^3) and bond valence sums (BVS) for $(C_5H_{6.16}N_2Cl_{0.84})(IO_2Cl_2)$.	S6
Table S4	Hydrogen bond lengths (Å) for $(C_5H_{6.16}N_2Cl_{0.84})(IO_2Cl_2)$.	S7
Table S5	The assignments of the infrared absorption peaks for $(C_5H_{6.16}N_2Cl_{0.84})(IO_2Cl_2)$.	S7
Table S6	Angles between the crystallographic axes and the principal dielectric axes in the triclinic crystal $(C_5H_{6.16}N_2Cl_{0.84})(IO_2Cl_2)$.	S7
Figure S1	Crystals photograph of $(C_5H_{6.16}N_2Cl_{0.84})(IO_2Cl_2)$.	S8
Figure S2	Simulated and experimental powder X-ray diffraction patterns of $(C_5H_{6.16}N_2Cl_{0.84})(IO_2Cl_2)$.	S8
Figure S3	Energy dispersive spectrum of $(C_5H_{6.16}N_2Cl_{0.84})(IO_2Cl_2)$.	S9
Figure S4	SEM image and the elemental distribution maps of $(C_5H_{6.16}N_2Cl_{0.84})(IO_2Cl_2)$.	S9
Figure S5	TGA and DTA curves of $(C_5H_{6.16}N_2Cl_{0.84})(IO_2Cl_2)$ under the N_2 atmosphere.	S10
Figure S6	IR spectrum of $(C_5H_{6.16}N_2Cl_{0.84})(IO_2Cl_2)$.	S10
Figure S7	UV-vis-IR spectrum of $(C_5H_{6.16}N_2Cl_{0.84})(IO_2Cl_2)$.	S11
Figure S8	Luminescent spectra of 2-amino-5-chloropyridine and $(C_5H_{6.16}N_2Cl_{0.84})(IO_2Cl_2)$.	S11
Figure S9	The crystal orientation of $(C_5H_{6.16}N_2Cl_{0.84})(IO_2Cl_2)$ for birefringence measurement determined by single-crystal XRD.	S12
Figure S10	The crystal thickness of $(C_5H_{6.16}N_2Cl_{0.84})(IO_2Cl_2)$ used for birefringence measurement.	S12
Figure S11	The calculated band structure of $(C_5H_{6.16}N_2Cl_{0.84})(IO_2Cl_2)$.	S13
Figure S12	The partial and total density of states for $(C_5H_{6.16}N_2Cl_{0.84})(IO_2Cl_2)$.	S13
Figure S13	The calculated birefringence and refractive indices of $(C_5H_{6.16}N_2Cl_{0.84})(IO_2Cl_2)$.	S14
Figure S14	Electron density difference map for $(C_5H_{6.16}N_2Cl_{0.84})(IO_2Cl_2)$.	S14
Reference		S15

Section S1

Instruments and property characterizations

Powder X-ray diffraction (PXRD) pattern of $(C_5H_{6.16}N_2Cl_{0.84})(IO_2Cl_2)$ was performed on a Rigaku MiniFlex II diffractometer with graphite-monochromated Cu Kα radiation in the 2θ range of 5°-70° utilizing a scanning step width of 0.02°. Elemental analysis of C, N and H elements was tested on the EuroEA3000 elemental analyzer. The EDS analysis was measured on a field-emission scanning electron microscope (FESEM, Thermo Scientific Apreo 2 S) equipped with an energy-dispersive X-ray spectroscope (EDS, ANAX-30P-B). Thermogravimetric analysis (TGA) and differential thermal analysis (DTA) were implemented with a NETZCH STA 449F3 using a heating rate of 2 °C/min under a N_2 atmosphere. Infrared (IR) spectrum of $(C_5H_{6.16}N_2Cl_{0.84})(IO_2Cl_2)$ was recorded on a Magna 750 FT-IR spectrometer in the form of KBr pellets ranging from 4000 to 400 cm^{-1}. Ultraviolet-visible-near infrared (UV-vis-NIR) spectrum ranging from 2000 to 200 nm was recorded on a PerkinElmer Lambda 950 UV-vis-NIR spectrophotometer. By applying *Kubelka-Munk* function,[1] reflectance spectrum was converted into absorption spectrum. Steady state photoluminescence excitation spectra and emission spectra were collected on a spectrometer equipped with continuous (450 W) xenon lamp (FLS1000, Edinburgh Instrument). The absolute photoluminescence quantum yield was obtained on a FLS1000 spectrometer mounted with a barium sulfate-coated integrating sphere as the sample chamber, in which the spectral data were corrected for the spectral response of both the spectrometer and the integrating sphere. The birefringence of $(C_5H_{6.16}N_2Cl_{0.84})(IO_2Cl_2)$ was measured on the small single crystal using the (010) crystal plane by a ZEISS Axio A1 polarizing microscope with a Berek compensator. The formula for calculating the birefringence is $R = d \times \Delta n$ (R denotes the optical path difference and d symbolizes the thickness).

Synthesis

Transparent single crystals of $(C_5H_{6.16}N_2Cl_{0.84})(IO_2Cl_2)$ were successfully grown by evaporation method in aqueous hydrochloric acid media. 2-aminopyridine (99%), iodic acid (99%) and HCl (37%) were purchased from Adamas. The HCl solution (1.67 mol/L) was obtained by diluting the purchased 37% HCl solution. The starting materials were 2-aminopyridine ($C_5H_6N_2$, 0.9412 g, 10 mmol), iodic acid (HIO_3, 3.5182 g, 20 mmol) and HCl (1.67 mol/L, 36 mL). The mixture of the starting materials was stirred for one hour and the resultant solution was allowed to evaporate at room temperature for two weeks. The clubbed yellowish crystals of $(C_5H_{6.16}N_2Cl_{0.84})(IO_2Cl_2)$ were obtained in a yield of 60% (based on the organic materials). The concentration of HCl solution should not be too high, otherwise $(ICl_2)^-$ and $(ICl_4)^-$ will be formed. The molar ratio of HCl/HIO_3 required for the synthesis is 3:1. Due to the large excess of HCl, partial iodic acid and concentrated hydrochloric acid undergo oxidation-reduction reaction to generate Cl_2. The 2-aminopyridine and Cl_2 undergo electrophilic substitution and protonate with H^+ cations to form $(C_5H_{6.16}N_2Cl_{0.84})^+$ cations. One H atom on the meta position of 2-aminopyridine is partially replaced by Cl(3) atom with Cl/H ratio of 0.84 : 0.16. Anal. Cald. for $C_5H_{6.16}N_2O_2Cl_{2.84}I$ (%): C 16.97, N 7.92, H 1.76. Found (%): C 16.92, N 7.73, H 2.05.

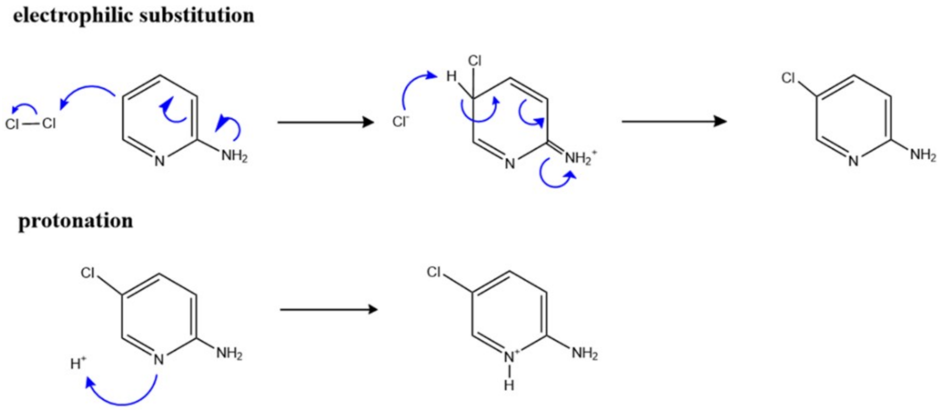

Single crystal structure determination

Single-crystal X-ray diffraction data of $(C_5H_{6.16}N_2Cl_{0.84})(IO_2Cl_2)$ were collected on a Rigaku Oxford Diffraction XtaLAB Synergy-R system with Mo Kα radiation (λ = 0.71073 Å) at 294 K. Data reduction was accomplished with CrysAlisPro, and absorption correction based on the multi-scan method was applied.[2] The structure was solved with the ShelXT 2014/5 solution program using Intrinsic Phasing methods and by utilizing Olex 2 as the graphical interface.[3,4] The

structure was refined with ShelXL 2018/3 using full matrix least squares minimization on F^2.[5] In $(C_5H_{6.16}N_2Cl_{0.84})(IO_2Cl_2)$, the thermal displacement parameter of the Cl(3) atom attached to the pyridine ring is high, and the occupancy factor was refined to be 84.036% for Cl(3), indicating the mixed occupancy of Cl and H at this atomic site. Examples of halogen atom and the H atom showing mixed occupancy have also been reported previously, indicating that it is reasonable.[6] All of the non-hydrogen atoms were refined anisotropically. The H atoms were located at geometrically calculated positions and refined with isotropic thermal parameters. The structure was checked for missing symmetry elements using PLATON, and none was found.[7] Crystallographic data and structural refinements of $(C_5H_{6.16}N_2Cl_{0.84})(IO_2Cl_2)$ are listed in Table S1, and the selected bond distances are listed in Table S2.

Computational methods

Calculations of electronic structure and optical property for $(C_5H_{6.16}N_2Cl_{0.84})(IO_2Cl_2)$ were performed utilizing CASTEP based on density function theory (DFT).[8-9] Norm-conserving pseudopotential was used to treat the electron-core interactions, and GGA-PBE was chosen as exchange-correlation function.[10-11] The following orbital electrons were treated as valence electrons: I-$5s^25p^5$, Cl-$3s^23p^5$, C-$2s^22p^2$, N-$2s^22p^3$, O-$2s^22p^4$, and H-$1s^1$. The numbers of plane waves included in the basis sets were determined by a cutoff energy of 750 eV for $(C_5H_{6.16}N_2Cl_{0.84})(IO_2Cl_2)$. Monkhorst-Pack k-point sampling of 4 × 3 × 3 was applied to perform numerical integration of Brillouin zone for $(C_5H_{6.16}N_2Cl_{0.84})(IO_2Cl_2)$. During the optical property calculations, more than 2 times of valence bands for $(C_5H_{6.16}N_2Cl_{0.84})(IO_2Cl_2)$ were applied to ensure the convergence of linear optical property. To further compare the optical properties of the conventional functional groups and the $(IO_2Cl_2)^-$ group, their electronic structures in molecular level were calculated through DFT method implemented by Gaussian16 package at the B3LYP.[12]

The optical permittivity tensor matrix of $(C_5H_{6.16}N_2Cl_{0.84})(IO_2Cl_2)$ was calculated and converted to the principal axes transformation. The calculations and analyses of optical property for $(C_5H_{6.16}N_2Cl_{0.84})(IO_2Cl_2)$ was based on the principal dielectric axis coordinate system.

$$\begin{bmatrix} 2.76157 & 0.00179 & -0.14664 \\ 0.00179 & 2.53553 & 0.05255 \\ -0.14664 & 0.05255 & 3.83380 \end{bmatrix} \xrightarrow{\text{principal axes transformation}} \begin{bmatrix} 2.74219 & 0 & 0 \\ 0 & 2.53313 & 0 \\ 0 & 0 & 3.85560 \end{bmatrix}$$

We defined the polarizability anisotropy-weighed electron density (PAWED) method to elucidate the optical anisotropy (birefringence) of $(C_5H_{6.16}N_2Cl_{0.84})(IO_2Cl_2)$ in a directly-perceived visual image:

$$\rho_{\Delta\chi}^{occ}(r) = \sum_i^{occ} \omega_i |\psi_i(r)|^2$$

$$\rho_{\Delta\chi}^{unocc}(r) = \sum_i^{unocc} \omega_i |\psi_i(r)|^2$$

Herein, $\rho_{\Delta\chi}^{occ}(r)$ and $\rho_{\Delta\chi}^{unocc}(r)$ are the electron density distributions of the polarizability anisotropy in the valence bands and conduction bands, respectively; $|\psi_i(r)|^2$ is the electron density of the *i*th band/orbital; ω_i is weighting factor, which can be formulized as

$$\omega_i = \frac{\Delta\chi_i}{\Delta\chi_{total}} = \frac{\varepsilon_i(zz) - \varepsilon_i(xx)}{\varepsilon(zz) - \varepsilon(xx)}$$

We used the PAWED method to reveal the structural origin of the birefringence. The contributed values of atoms/ions/groups to the polarizability anisotropy can also be calculated. First, the electron density of the compound is cut in the real space, concretely, the electron density of each grid point is assigned to the corresponding atom by using kinds of cutting methods, e.g. gradient path interpolation tracing (GPIT) method, etc. Then based on the polarizability anisotropy-weighted electron density (PAWED) data, the polarizability anisotropy of all the grid points belonging to a certain atom are summed up to obtain the polarizability anisotropy contribution value of the atom. Finally, the birefringence contribution of a group can be further obtained by summing up those of all atoms in the group.

Table S1. Crystallographic data for $(C_5H_{6.16}N_2Cl_{0.84})(IO_2Cl_2)$.

formula	$C_5H_{6.16}N_2O_2Cl_{2.84}I$
formula weight	353.86
crystal system	triclinic
space group	*P*-1
T (K)	294(2)
a (Å)	7.5387(5)
b (Å)	8.2803(5)
c (Å)	8.4979(5)
α (deg)	83.344(5)
β (deg)	89.699(5)
γ (deg)	70.311(6)
V (Å^3)	495.76(6)
Z	2
λ (Mo-K*α*) (Å)	0.71073
D_c (Mg/m^3)	2.370
μ (mm^{-1})	3.962
goodness of fit on F^2	1.040
R_1, wR_2 [I >2σ(I)][a]	0.0239, 0.0512
R_1, wR_2 (all data)[a]	0.0290, 0.0536

[a] $R_1 = \sum||F_o| - |F_c||/\sum|F_o|$, and $wR_2 = \{\sum w[(F_o)^2 - (F_c)^2]^2/\sum w[(F_o)^2]^2\}^{1/2}$.

Table S2. Selected bond lengths (Å) for ($C_5H_{6.16}N_2Cl_{0.84}$)(IO_2Cl_2).

Bond	Bond lengths
I(1)-Cl(1)	2.4963(8)
I(1)-Cl(2)	2.4929(8)
I(1)-O(1)	1.784(2)
I(1)-O(2)	1.791(2)
Cl(3)-C(2)	1.715(4)
N(1)-C(1)	1.360(4)
N(1)-C(5)	1.346(4)
N(2)-C(5)	1.325(4)
C(1)-C(2)	1.359(5)
C(2)-C(3)	1.399(5)
C(3)-C(4)	1.344(5)
C(4)-C(5)	1.410(5)

Table S3. Atomic coordinates (×10^4), equivalent isotropic displacement parameters (Å^2 ×10^3) and bond valence sums (BVS) for ($C_5H_{6.16}N_2Cl_{0.84}$)(IO_2Cl_2).

Atom	x	y	z	U(eq)	BVS
I(1)	2601(1)	9746(1)	137(1)	21(1)	5.048
Cl(1)	2302(1)	9747(1)	-2788(1)	38(1)	0.730
Cl(2)	2719(1)	10149(1)	2991(1)	36(1)	0.737
Cl(3)	1118(2)	5505(2)	8208(1)	51(1)	/
O(1)	4949(3)	8241(3)	228(3)	30(1)	1.807
O(2)	1330(3)	8276(3)	554(2)	29(1)	1.774
N(1)	1904(4)	6306(4)	3639(3)	30(1)	/
N(2)	3610(5)	4623(4)	1800(3)	41(1)	/
C(1)	1283(5)	6599(5)	5120(4)	33(1)	/
C(2)	1912(6)	5291(5)	6323(4)	36(1)	/
C(3)	3201(6)	3702(5)	6019(4)	37(1)	/
C(4)	3783(5)	3443(5)	4543(4)	34(1)	/
C(5)	3102(5)	4785(4)	3284(4)	28(1)	/

Table S4. Hydrogen bond lengths (Å) for ($C_5H_{6.16}N_2Cl_{0.84}$)(IO_2Cl_2).

D—H…A	d(D-H)	d(H…A)	d(D…A)	∠(D-H…A)(°)
N(1)-H(1)…O(2)	0.86	2.08	2.875(3)	154.3
N(2)-H(2A)…O(2)	0.86	2.25	2.999(4)	145.2
N(2)-H(2B)…O(1)#1	0.86	2.25	2.994(4)	145.3

Symmetry transformations used to generate equivalent atoms: #1 -x+1, -y+1, -z.

Table S5. The assignments of the infrared absorption peaks for ($C_5H_{6.16}N_2Cl_{0.84}$)(IO_2Cl_2).

IR peak (cm^{-1})	Assignment
3460-3220	N-H stretching bands
3220-2930	C-H stretching bands
1710-1480	C-C and C-N stretching bands
1480-1350	N-H in-plane bending bands
1350-1180	C-H in-plane bending bands
1180-1188	N-H out-plane bending bands
1011-890	C-H out-plane bending bands
890-650	C-Cl stretching bands
890-650	I-Cl and I-O stretching bands
650-400	I-Cl and I-O bending bands

Table S6. Angles between the crystallographic axes and the principal dielectric axes in the triclinic crystal ($C_5H_{6.16}N_2Cl_{0.84}$)(IO_2Cl_2).

∠	*a*-O-*x*	*b*-O-*x*	*c*-O-*x*	*a*-O-*y*	*b*-O-*y*	*c*-O-*y*	*a*-O-*z*	*b*-O-*z*	*c*-O-*z*
angle (deg)	18.765	86.744	82.470	72.326	9.436	92.547	96.105	81.154	7.954

The crystallographic axes: *a*, *b* and *c*; the principal dielectric axes: *x*, *y*, and *z*.

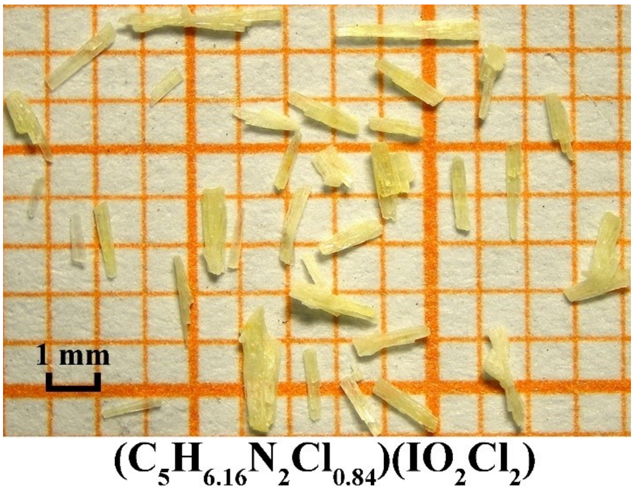

Figure S1. Crystals photograph of $(C_5H_{6.16}N_2Cl_{0.84})(IO_2Cl_2)$.

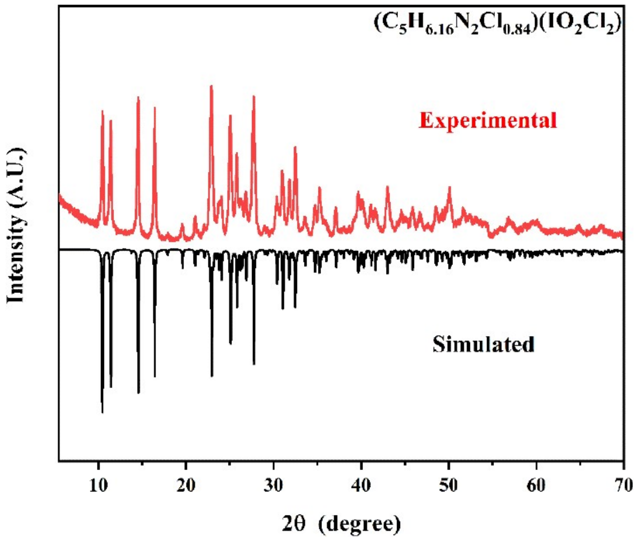

Figure S2. Simulated and experimental powder X-ray diffraction patterns of $(C_5H_{6.16}N_2Cl_{0.84})(IO_2Cl_2)$.

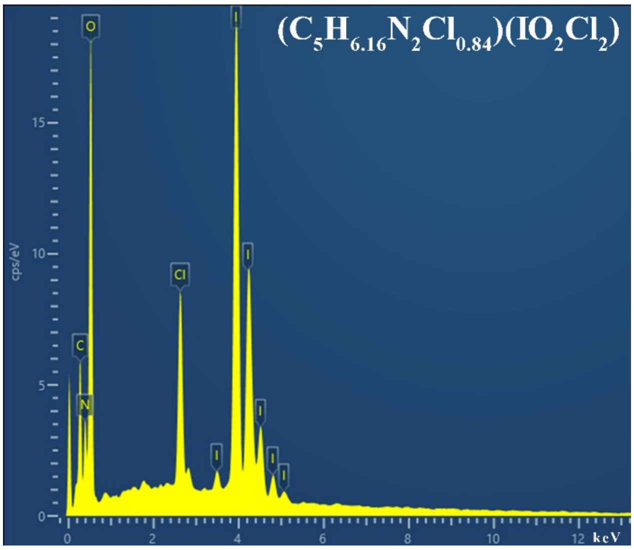

Figure S3. Energy dispersive spectrum of $(C_5H_{6.16}N_2Cl_{0.84})(IO_2Cl_2)$.

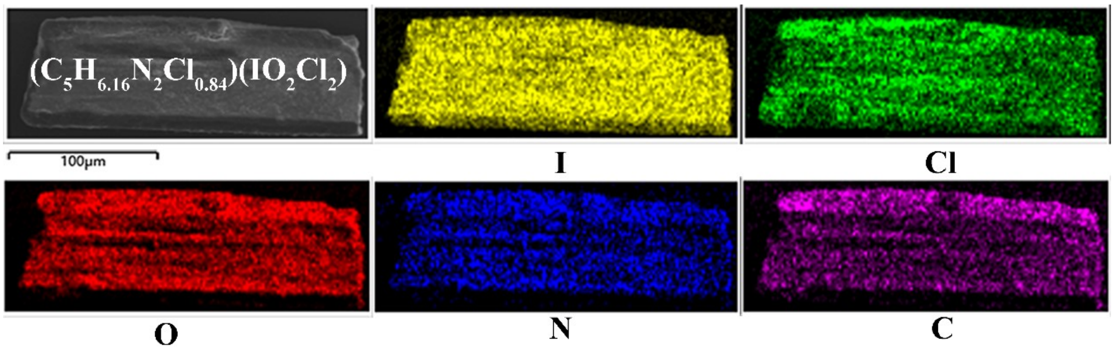

Figure S4. SEM image and the elemental distribution maps of $(C_5H_{6.16}N_2Cl_{0.84})(IO_2Cl_2)$.

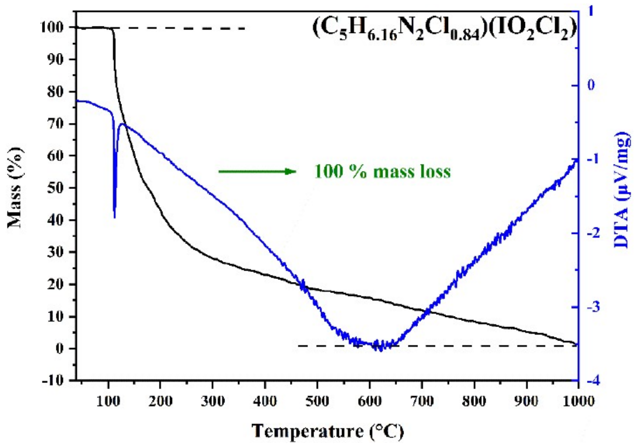

Figure S5. TGA and DTA curves of $(C_5H_{6.16}N_2Cl_{0.84})(IO_2Cl_2)$ under the N_2 atmosphere.

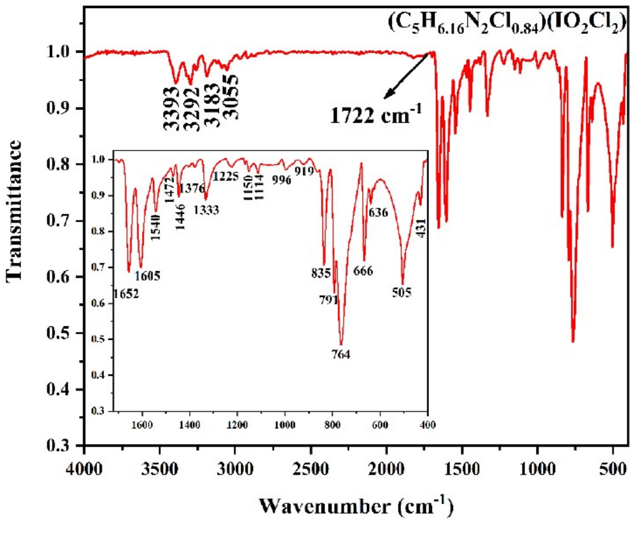

Figure S6. IR spectrum of $(C_5H_{6.16}N_2Cl_{0.84})(IO_2Cl_2)$.

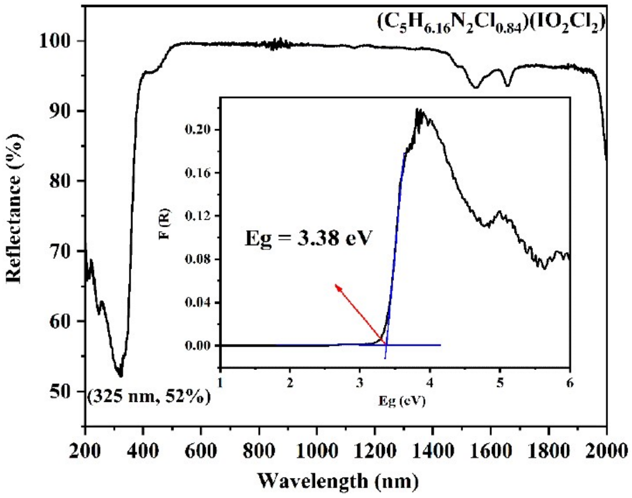

Figure S7. UV-vis-IR spectrum of $(C_5H_{6.16}N_2Cl_{0.84})(IO_2Cl_2)$.

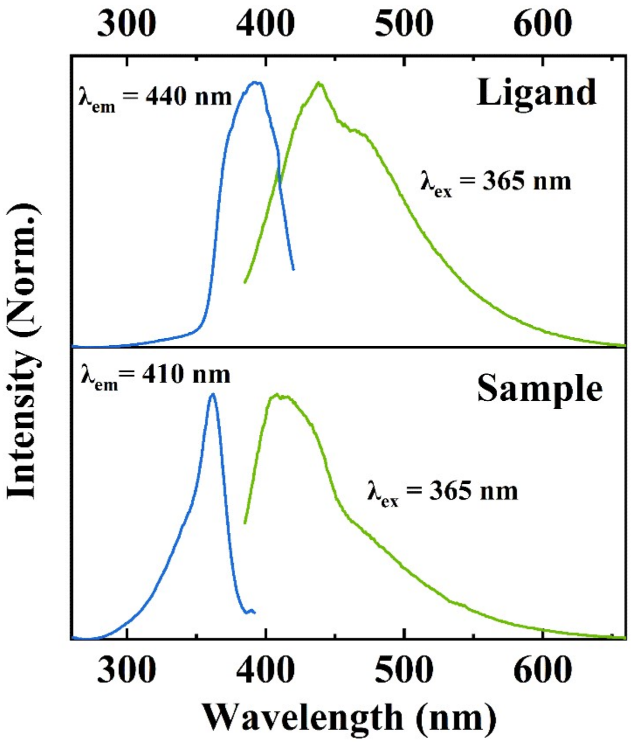

Figure S8. Luminescent spectra of 2-amino-5-chloropyridine and $(C_5H_{6.16}N_2Cl_{0.84})(IO_2Cl_2)$.

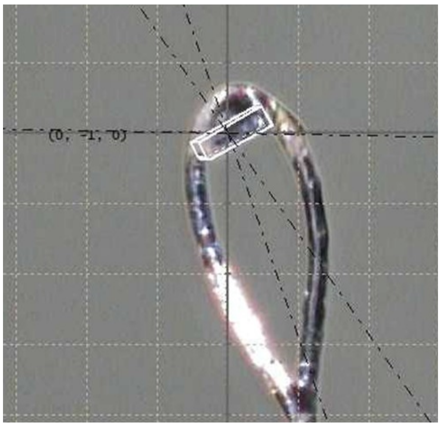

Figure S9. The crystal orientation of $(C_5H_{6.16}N_2Cl_{0.84})(IO_2Cl_2)$ for birefringence measurement determined by single-crystal XRD.

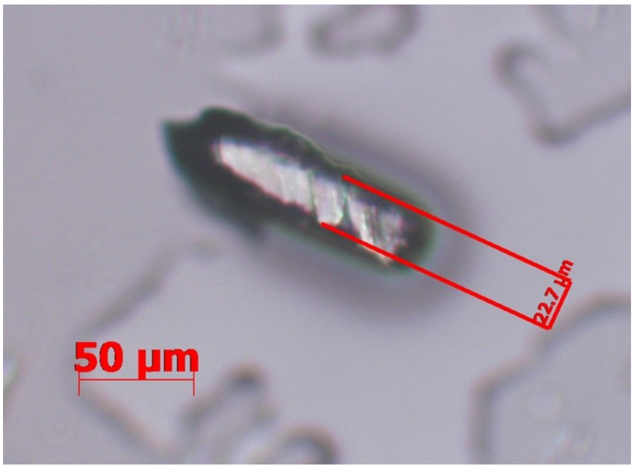

Figure S10. The crystal thickness of $(C_5H_{6.16}N_2Cl_{0.84})(IO_2Cl_2)$ used for birefringence measurement.

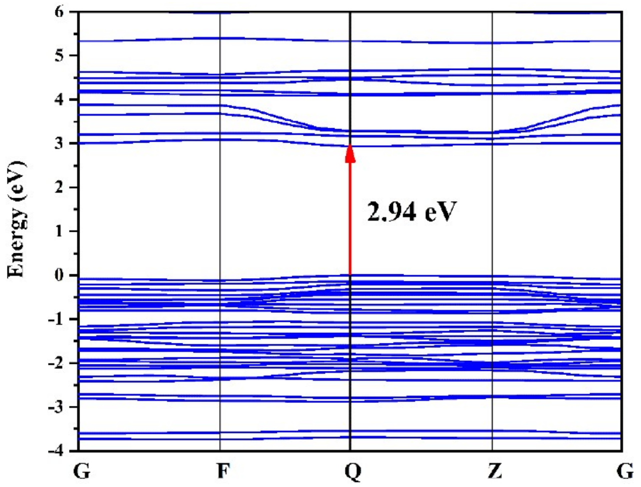

Figure S11. The calculated band structure of $(C_5H_{6.16}N_2Cl_{0.84})(IO_2Cl_2)$.

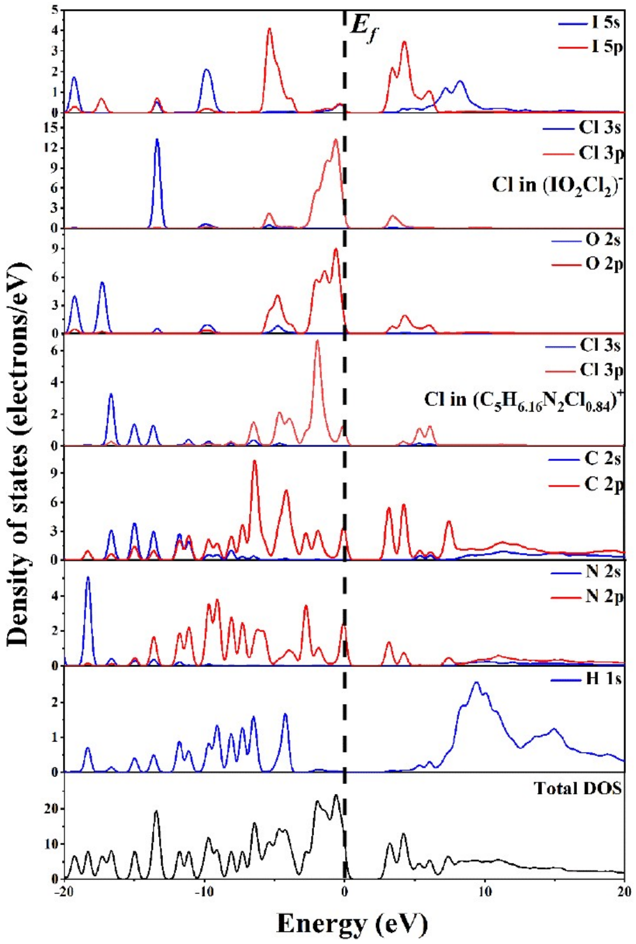

Figure S12. The partial and total density of states for ($C_5H_{6.16}N_2Cl_{0.84}$)(IO_2Cl_2).

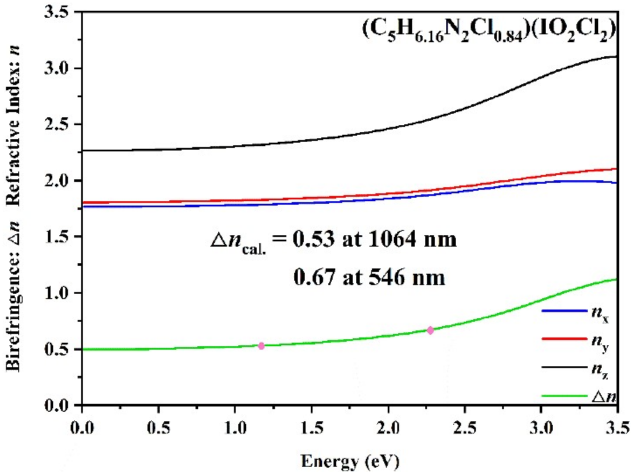

Figure S13. The calculated birefringence and refractive indices of ($C_5H_{6.16}N_2Cl_{0.84}$)(IO_2Cl_2).

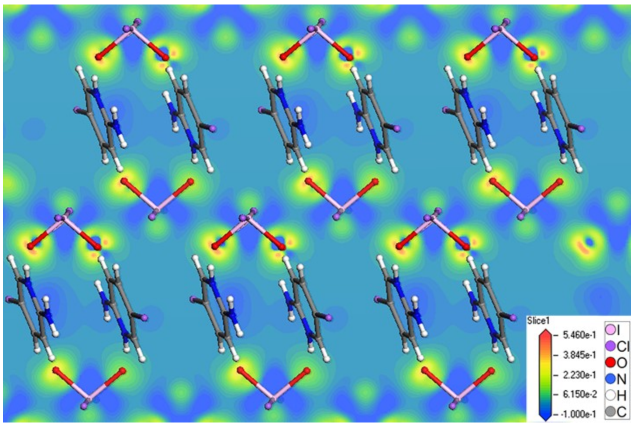

Figure S14. Electron density difference map for ($C_5H_{6.16}N_2Cl_{0.84}$)(IO_2Cl_2).

References

1. P. Kubelka and F. Munk, An Article on Optics of Paint Layers, *Z. Tech. Phys.*, 1931, **12**, 259-274.
2. R. H. Blessing, An Empirical Correction for Absorption Anisotropy, *Acta Crystallogr., Sect. A: Found. Crystallogr.*, 1995, **51**, 33-38.
3. G. M. Sheldrick, SHELXT - Integrated Space-Group and Crystal-Structure Determination, *Acta Crystallogr., Sect. A: Found. Adv.,* 2015*,* **71***,* 3-8*.*
4. O. V. Dolomanov, L. J. Bourhis, R. J. Gildea, J. A. K. Howard and H. Puschmann, *OLEX2*: A Complete Structure Solution, Refinement and Analysis Program, *J. Appl. Crystallogr.*, 2009, **42**, 339-341.
5. G. M. Sheldrick, Crystal Structure Refinement with SHELXL, *Acta Crystallogr., Sect. C: Struct. Chem.,* 2015*,* **71***,* 3-8*.*
6. M. Keikha, M. Pourayoubi, J. P. Jasinski and J. A. Golen, Bis(pyrrolidin-1-yl)phosphinic (2,4-difluorobenzoyl)amide, *Acta Crystallogr., Sect. E*, 2012, **68**, o2688.
7. A. L. Spek, Single-Crystal Structure Validation with the Program PLATON, *J. Appl. Crystallogr.* 2003, **36**, 7-13.
8. M. D. Segall, P. J. D. Lindan, M. J. Probert, C. J. Pickard, P. J. Hasnip, S. J. Clark and M. C. Payne, First-Principles Simulation: Ideas, Illustrations and the CASTEP Code, *J. Phys.: Condens. Mat.*, 2002, **14**, 2717-2744.
9. V. Milman, B. Winkler, J. A. White, C. J. Pickard, M. C. Payne, E. V. Akhmatskaya and R. H. Nobes, Electronic Structure, Properties, and Phase Stability of Inorganic Crystals: A Pseudopotential Plane-Wave Study, *Int. J. Quantum Chem.*, 2000, **77**, 895-910.
10. J. P. Perdew, K. Burke and M. Ernzerhof, Generalized Gradient Approximation Made Simple, *Phys. Rev. Lett.*, 1996, **77**, 3865-3868.
11. J. S. Lin, A. Qteish, M. C. Payne and V. Heine, Optimized and Transferable Nonlocal Separable *ab initio* Pseudopotentials, *Phys. Rev. B: Condens. Matter Mater. Phys.*, 1993, **47**, 4174-4180.
12. T. Lu and F. W. Chen, Multiwfn: A Multifunctional Wavefunction Analyzer, *J. Comput. Chem.*, 2012, **33**, 580-592.
